# Evolution of a Planktonic Foraminifer during Environmental Changes in the Tropical Oceans

**DOI:** 10.1371/journal.pone.0148847

**Published:** 2016-02-17

**Authors:** Yurika Ujiié, Yoshiyuki Ishitani

**Affiliations:** 1 Department of Biology, Shinshu University, Matsumoto, Nagano, Japan; 2 Atmosphere and Ocean Research Institute, The University of Tokyo, Kashiwa, Chiba, Japan; Field Museum of Natural History, UNITED STATES

## Abstract

Ecological adaptation to environmental changes is a strong driver of evolution, enabling speciation of pelagic plankton in the open ocean without the presence of effective physical barriers to gene flow. The tropical ocean environment, which plays an important role in shaping marine biodiversity, has drastically and frequently changed since the Pliocene. Nevertheless, the evolutionary history of tropical pelagic plankton has been poorly understood, as phylogeographic investigations are still in the developing state and paleontological approaches are insufficient to obtain a sequential record from the deep-sea sediments. The planktonic foraminifer *Pulleniatina obliquiloculata* is widely distributed in the tropical area throughout the world’s oceans, and its phylogeography is well established. It is thus one of the best candidates to examine how past environmental changes may have shifted the spatial distribution and affected the diversification of tropical pelagic plankton. Such an examination requires the divergence history of the planktonic foraminifers, yet the gene marker (partial small subunit (SSU) rDNA) previously used for phylogeographic studies was not powerful enough to achieve a high accuracy in estimating the divergence times. The present study focuses on improving the precision of divergence time estimates for the splits between sibling species (genetic types) of planktonic foraminifers by increasing the number of genes as well as the number of nucleotide bases used for molecular clock estimates. We have amplified the entire coding regions of two ribosomal RNA genes (SSU rDNA and large subunit (LSU) rDNA) of three genetic types of *P*. *obliquiloculata* and two closely related species for the first time and applied them to the Bayesian relaxed clock method. The comparison of the credible intervals of the four datasets consisting either of sequences of the partial SSU rDNA, the complete SSU rDNA, LSU rDNA, or a combination of both genes (SSU+LSU) clearly demonstrated that the two-gene dataset improved the accuracy of divergence time estimates. The *P*. *obliquiloculata* lineage diverged twice, first at the end of the Pliocene (3.1 Ma) and again in the middle Pleistocene (1.4 Ma). Both timings coincided with the environmental changes, which indirectly involved geographic separation of populations. The habitat of *P*. *obliquiloculata* was expanded toward the higher latitudinal zones during the stable warm periods and subsequently placed on the steep environmental gradients following the global cooling. Different environmental conditions in the stable warm tropics and unstable higher latitudes may have triggered ecological divergence among the populations, leading to adaptive differentiation and eventually speciation. A comprehensive analysis of divergence time estimates combined with phylogeography enabled us to reveal the evolutionary history of the pelagic plankton and to find the potential paleoenvironmental events, which could have changed their biogeography and ecology.

## Introduction

Understanding the evolution of pelagic plankton during the drastic environmental changes of the past few million years is crucial for estimating the effects of global changes on future biodiversity. The pelagic plankton, particularly unicellular plankton, are known to disperse passively and widely because of the little motility during their entire life-cycles and the absence of geographic barriers in the open ocean (e.g. [1,2]). However, the phylogeographic studies have unexpectedly revealed the genetic differentiations and the limited dispersal among the geographically distant populations in the pelagic unicellular plankton [3,4,5]. Moreover, a recent study has demonstrated that speciation of pelagic protist occurred after differential adaptation of two sister species to trophic niches separated vertically in the water column [6]. Theoretically, pelagic plankton should be able to evolve rapidly in response to the environmental changes; however, their adaptation to a specific environment is still little understood [7]. Ecological differences between environments cause divergent selection that may lead to changes in various traits of individuals [8]. If these traits control reproductive isolation, adaptive divergence in those traits may reduce gene flow [8]. In the open ocean, therefore, the adaption to distinct environments can constrain gene flow without the split of populations by physical barriers. A combined analysis of divergence time estimate and phylogeography can help to find the tectonic and climatic events that likely may have changed the geographic distribution of pelagic plankton and their ecological characters. Thus, in the present study, we have attempted to determine whether any physical (geographic) separation of populations or rather ecological divergence related to the environmental changes is more effective in impeding the gene flow of pelagic plankton.

The tropical oceans are a biodiversity hotspot for both coastal and pelagic organisms [2,9–11]. In particular, the tropical coasts of the Indo-Pacific Oceans have been a marine hotspot since the Miocene [10]. These warm environments expanded in latitudinal direction from the middle Miocene to Pliocene [12,13]. However, this warm state cooled around ~4 million years ago (Ma) toward the Pleistocene owing to the geographic separations between the tropical Pacific and Atlantic Oceans [14] and the tropical Indian and Pacific Oceans [15], as well as to the atmospheric-oceanic environmental changes [16–18]. The modern geographic and climatic conditions have been established after the Pleistocene, when the large glacial/interglacial cycles started [16,17]. The dispersal and biogeographic patterns of the tropical coastal organisms changed, leading to speciation during the Plio-Pleistocene environmental developments, such as geographic connections and separations between the ocean basins [19,20]. However, little is known about the evolution of the tropical pelagic plankton that could be associated with the biogeographic and ecological changes in the past few million years.

**Fig 1:**
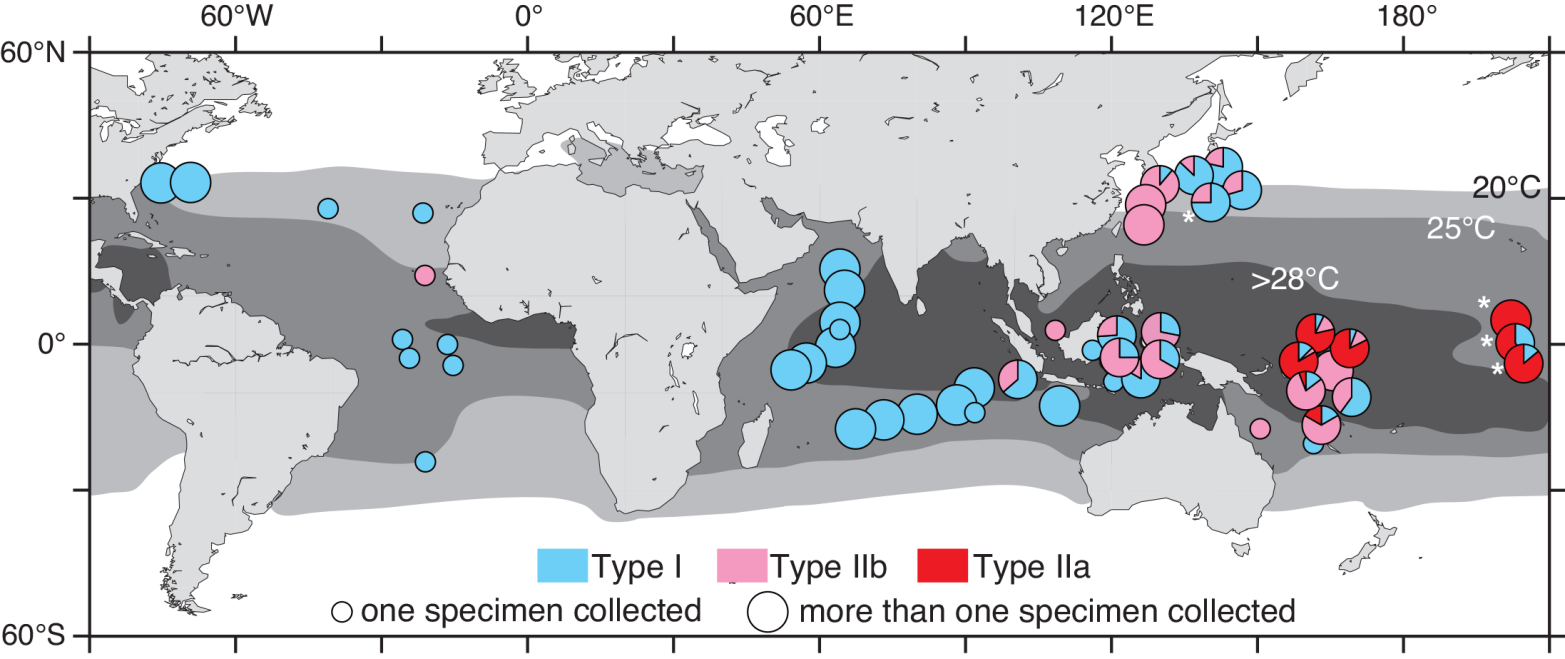
Geographic distribution of the three genetic types of *P*. *obliquiloculata*. Pie chart shows the frequencies of three genetic types at each sampling site based on the previous studies [5,28]. The small and large circles indicate the locations where only one or more specimens were collected. The asterisks show the locations, where the samples were obtained for the present study. The genetic types of *P*. *obliquiloculata* specimens from Seears et al. [28] were identified based on the alignment of the partial SSU rDNA sequences with all existing data of Ujiié et al. [5]. Grey areas on the map show the ranges of water temperatures between 20–25, 25–28, and >28°C. The temperature data were obtained from the World Ocean Atlas 2005 [29]. The map is drawn by using the Ocean Data View [30].

Among pelagic plankton, the biodiversity of planktonic foraminifers has been well studied. They have been examined using the ribosomal gene marker [21] and are known to have one of the best fossil records since their appearance in the Jurassic [22]. The extant morphospecies, which are classified according to morphological differences of their calcareous shells, have appeared from the Miocene or Pliocene [23]. These morphospecies contain multiple genetically isolated species (i.e. genetic types; compiled in [21]), and almost all the existing genetic types are monophyletic within the morphospecies (e.g. [24,25]). Therefore, the divergences in these genetic types must have occurred after the appearance of the morphospecies lineage (after the Miocene). The morphospecies *Pulleniatina obliquiloculata* is distributed from the tropical to subtropical areas in the world’s oceans since its first appearance at the end of the Miocene (~5.8 Ma) [23] and it is abundant in the tropical Indo-Pacific Oceans [26,27]. This species is a powerful candidate for examining the relationship between evolution and environmental changes in the tropical pelagic oceans. However, the sole use of fossils is insufficient in tracing the evolutionary history, as most of the tropical ocean basin is deeper than the carbonate compensation depth wherein the carbonates of foraminiferal shells are dissolved. Recently, the molecular phylogeographic studies demonstrated that the morphospecies *P*. *obliquiloculata* has three genetic types (Types I, IIa, and IIb) with distinctive distribution in the world’s oceans [5,28] (Fig 1). Estimating the divergence times of these genetic types enables us now to understand the biodiversity responses to the environmental changes in the tropical oceans during the Plio-Pleistocene.

The molecular clock concept based on the substitution rates of nucleotide and amino acid sequences is useful for estimating the divergence time between the lineages. The divergence times of the genetic types of planktonic foraminifers were initially estimated using a strict molecular clock, in which the substitution rate was assumed to be the same among the lineages [31]. However, since the nucleotide substitution rates vary greatly among the planktonic foraminiferal lineages [32], this assumption introduced bias in the estimation of divergence time. Subsequently, the use of algorithms based on maximum likelihood (ML) and Bayesian frameworks improved the estimation of divergence for lineages with variable evolutionary rates (e.g. [33]). Recent studies have employed relaxed clocks to estimate the timing of genetic divergence in planktonic foraminifers [24,34,35], though the credible intervals (CI) in these estimations are still too large. Such large intervals (>3 million years) overlap with multiple geological events in the Plio-Pleistocene and obscure the relationships between the divergence times and specific environmental changes. All the previous molecular clock analyses used the partial SSU rDNA sequences. Although this ~1000 base pair (bp) fragment (one-third of the entire length) contains useful variable regions (helices between 34 and 44) to identify the foraminiferal species and genetic types [21,36], it is difficult to achieve a precise estimation of the divergence time with such a short fragment. While the evolutionary rates vary among genes [37], the use of multiple genes can compensate this issue. Multiple gene markers like par actin-2, β-tubulin, and RPB1 are suitable markers for estimating the phylogeny at the family or higher levels of foraminifers [38,39]. Groussin et al. [40] used these genes in combination with the SSU rDNA to improve and succeed in estimating the divergence time with better accuracy for the early evolution of foraminiferal orders. Increasing the number of nucleotide bases and genes, which have enough resolution to show the phylogenetic relationship of the genetic types, facilitates more precise estimation of their divergence times in the past few million years. Thus, as the SSU rDNA and LSU rDNA have been used for phylogenetic analyses at the species level of foraminifers [41], we employed these genes to improve the precision of the divergence time estimation of the genetic types of *P*. *obliquiloculata*.

In the present study, we sequenced the entire coding regions of SSU rDNA and LSU rDNA from three morphospecies of planktonic foraminifers for the first time and investigated the phylogenetic relationships among the three genetic types of *P*. *obliquiloculata*. The divergence times estimated by using the Bayesian relaxed clock method based on different gene-datasets and fossil calibration sets were compared to examine the effects of increasing the number of nucleotide bases and different constraints on estimation. Then, we inferred a possible evolutionary scenario for *P*. *obliquiloculata* with respect to the environmental changes in the tropical ocean during the Plio-Pleistocene.

## Materials and Methods

Nine *P*. *obliquiloculata* specimens derived from the samples that were previously collected at the three stations (St. C, D, and E) in the central Pacific equatorial area [5] were used in the present study (Fig 1). Four additional *P*. *obliquiloculata* specimens and two specimens each of *Neogloboquadrina dutertrei* and *Globorotalia inflata*, as outgroups, were newly collected from off Japan in the northwest Pacific. No specific permissions were required for the sampling location because it is the open sea. Our field studies did not involve endangered or protected species. All the examined specimens were individually cleaned in filtered seawater under a microscope.

### DNA Extraction, Amplification, Cloning, and Sequencing

**Fig 2:**
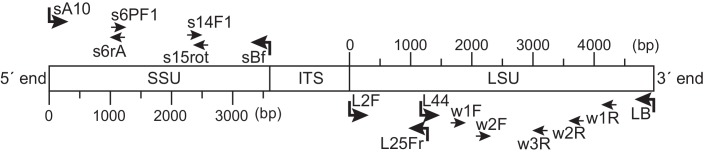
Location of the primers used to amplify and sequence the SSU rDNA and LSU rDNA. The names and orientations of the primers correspond to Table 1. The primers used for PCR amplification are shown by key arrows.

Genomic DNA was extracted from each specimen in accordance with the guanidinium thiocyanate protocol [5]. Nearly the entire coding region (~3500 bp) of SSU rDNA was amplified by polymerase chain reaction (PCR) analysis using the primers sA10 and sBf (Table 1). Two overlapping PCR-amplified fragments for LSU rDNA were assembled: the 5´ region (~1250 bp) was amplified using primers L2f and L25fr, and the 3´ region (~3500 bp) was amplified using primers L44 and LB (Table 1). The following PCR conditions were maintained: 40 cycles of 95°C (30 s)–50°C (40 s)–72°C (3 min) with a final elongation step of 10 min at 72°C for SSU rDNA, 40 cycles of 95°C (30 s)–52°C (40 s)–72°C (2 min) with a final elongation step of 10 min at 72°C for 5´-part of LSU rDNA, and 40 cycles of 95°C (30 s)–56°C (40 s)–72°C (3 min) with a final elongation step of 10 min at 72°C for 3´-part of LSU rDNA. The PCR products were cloned with a StrataClone PCR Cloning Kit (Agilent Technologies, CA, USA). This was followed by the sequencing of eight clones from each specimen by the primer walking method with internal oligonucleotide primers using the ABI Prism 3130 Genetic Analyzer (Applied Biosystems, CA, USA) at the Center for Advanced Marine Core Research, Kochi University (Table 1, Fig 2). All the examined clones from a single specimen contained the identical rDNA sequence without single nucleotide polymorphisms. In total, 15 SSU rDNA sequences and 17 LSU rDNA sequences were deposited in the GenBank (accession numbers LC049304–LC049335).

**Table 1:** Primers used for the PCR amplification and sequencing.

Primer	Sequence 5´-3´	Orientation	Reference
**SSU rDNA**			
sA10	CTCAAAGATTAAGCCATGCAAGTGG	Forward	[43]
*s6rA	GCACCAGACTTGCCC	Reverse	[43]
*s6PF1	GAGGCAGTGACAAGCTG	Forward	This study
*s15rot	CATAATCATGAAAGGACTAGC	Reverse	[43]
*s14F1	AAGGGCACCACAAGAACGC	Forward	[43]
sBf	TGATCCATC(AG)GCAGGTTCACCTAC	Reverse	[5]
**LSU rDNA**			
L2F	AGTAACGGCGAGTGA	Forward	This study
L25Fr	CTAGATGGTTCGATGAGTCTT	Reverse	This study
L44	ACCCGAAAGATGGTGAAC	Forward	[38]
*w1F	AAGCAGGACTGGCGATG	Forward	This study
*w2F	GAGCTATCCATAGTGCAG	Forward	This study
*w3R	CAGTCGGATTCCCCGAG	Reverse	This study
*w2R	ACTGCCTCTTACAATCTTCG	Reverse	This study
*w1R	AGCCAAACTCCCATTCTGC	Reverse	This study
LB	CGACGGTCTAAACCCAG	Reverse	[38]

* the internal primer for sequencing only.

### Phylogenetic Analysis

Each nucleotide sequence of the SSU rDNA and LSU rDNA was manually aligned with the SeaView v4.3.4 program [42]. Published sequences of *N*. *dutertrei* and *G*. *inflata* (GenBank: EU199449 and EU199447) [43], which were amplified by the same primers sA10 and sBf, were also included in the SSU rDNA sequence dataset. After the ambiguously aligned sites had been excluded, 2685 sites of SSU rDNA sequences and 3591 sites of LSU rDNA were available for the phylogenetic analyses. Three analyses were performed: two with a single-gene (SSU or LSU) dataset and one with a two-gene (SSU+LSU) dataset.

For each single-gene analysis, the best-fit nucleotide substitution model was selected using MrModelTest 2.3 [44] and Treefinder [45] programs. For the SSU dataset, the general time reversible (GTR) [46] model was used with a gamma (Γ) [47] distribution for variable rates and a proportion of invariant sites (I) [48]. For the LSU dataset, a GTR model + Γ distribution were used. For the SSU+LSU dataset, we used separate model conditions that involved the individual optimization of all parameters for each gene.

Bayesian analyses were conducted on the three datasets with each of the optimal models, using MrBayes v 3.1.2 program [49]. The Markov Chain Monte Carlo (MCMC) process was set to enable the simultaneous functioning of four chains (three heated and one cold). Two independent runs were conducted for 1.6 × 10^6^ (SSU), 1.1 × 10^6^ (LSU) and 1.1 × 10^6^ (SSU+LSU) generations. The trees and log-likelihood values were sampled at 100-generation intervals. The first 6 × 10^5^ (SSU) and 1 × 10^5^ (LSU and SSU+LSU) generations were excluded as burn-in. Pooled trees (1.0 × 10^6^ generations) were used to obtain the Bayesian posterior probabilities for each dataset. The ML analyses for each of the three datasets were performed using Treefinder [45], and the bootstrap support was based on 1000 replicates in each dataset.

### Bayesian Divergence Time Estimation

**Fig 3:**
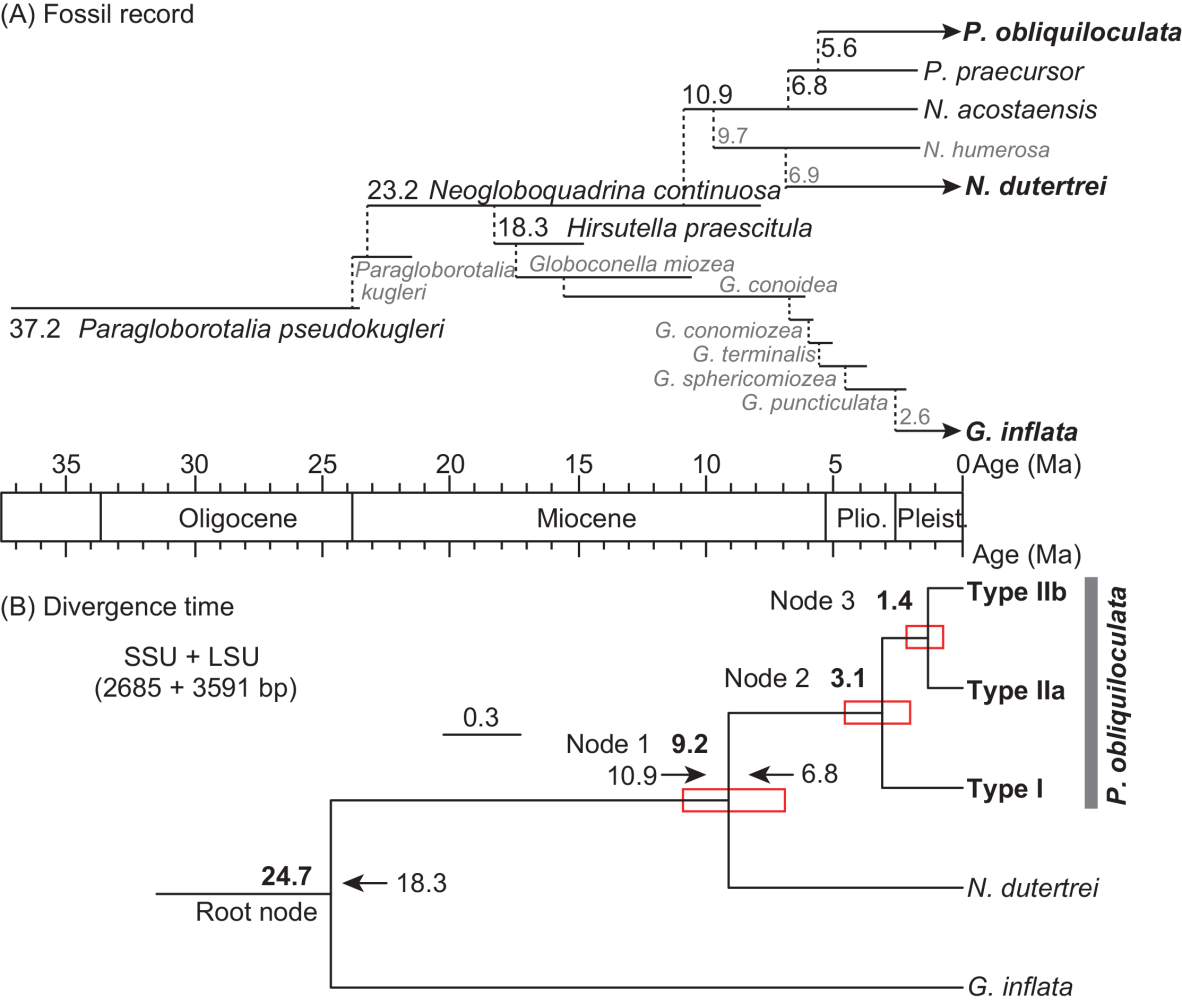
**(A) The hypothetical phylogeny of the studied morphospecies and their ancestors following the argument by Aze et al. [23].** Lines show the occurrence of each morphospecies, and the number indicates the FAD of each lineage. The black letters, lines, and numbers are used for the calibration sets shown in Table 2. Arrows indicate the extant species used for the present study. **(B) The phylogeny of *P*. *obliquiloculata* genetic types with divergence times estimated by using the SSU+LSU dataset with fossil calibration set-4.** The posterior mean divergence time (Ma) at each node is shown. The boxes enclosed by red lines indicate the 95% CIs.

The divergence times of five lineages (three genetic types of *P*. *obliquiloculata*, *N*. *dutertrei*, and *G*. *inflata*) were estimated by the Bayesian relaxed clock method, as implemented in the MCMCTREE program (PAML 4.4b) [50]. A representative sequence was selected from each of three *P*. *obliquiloculata* clades (T94, KH279, and KH281). In the further analyses, we compared the CIs among the partial SSU rDNA between the helices 34–44 (the region between the primers s14F1 and sBf in Fig 2), two single-genes (SSU rDNA and LSU rDNA), and the two-gene datasets. The differences in the divergence estimates were also examined using four fossil calibration sets (Table 2). The phylogenetic relationship of the fossils is inferred based on the morphological similarities of the shells (Fig 3A). Yet it is uncertain whether those morphological characters are inherited or not. Therefore, we considered four different combinations of calibration dates. The calibration set-1 used only the FAD of the extant species *P*. *obliquiloculata* for the minimum constraint of the node between *P*. *obliquiloculata* and *N*. *dutertrei* (Node 1) as the most relaxed constraint. The calibration set-2 employed the FAD of *Pulleniatina praecursor*, as the first divergence of the genus *Pulleniatina*, for the minimum constraint of Node 1. In accordance with the monophyletic origin of two lineages *P*. *obliquiloculata* and *N*. *dutertrei* as shown in the paleontological interpretation [23], the calibration set-3 added the FAD of common ancestor *Neogloboquadrina acostaensis* as the minimum constraint of the node between *P*. *obliquiloculata* + *N*. *dutertrei* and *G*. *inflata* (Root Node). Among the three extant species, *P*. *obliquiloculata*, *N*. *dutertrei*, and *G*. *inflata*, the lineage of *G*. *inflata* firstly diverged from the most common ancestor *Neogloboquadrina continuosa* (Fig 3A). Then, the tight constraint calibration set-4 used the FAD of *N*. *continuosa* as the minimum constraint of Root Node and the FADs of *N*. *acostaensis* and *P*. *praecursor* as the maximum and minimum constraints of Node 1, respectively.

**Table 2:** Upper (maximum) and Lower (minimum) constraints of the basal nodes used for estimating divergence dates.

Set #	Node #	Constraints (Age in Ma)	Appearance of morphospecies
1	1	L (5.6)	*Pulleniatina obliquiloculata*
2	1	L (6.8)	Pulleniatina praecursor*
3	root	L (10.9)	Neogloboquadrina acostaensis**
	1	L (6.8)	*Pulleniatina praecursor*
4	root	L (18.3)	Hirsutella praescitula***
	1	U (10.9)	*Neogloboquadrina acostaensis*
		L (6.8)	*Pulleniatina praecursor*

First appearance dates (FADs) as inferred by Aze et al. [23].

*Ancestral lineage of the genus *Pulleniatina*

**Common ancestor of *P*. *obliquiloculata* and *N*. *dutertrei*

*** Basal ancestral lineage of *G*. *inflata*

In all datasets, the ML estimates of branch lengths were obtained under the GTR + Γ model with the gamma priors set at 0.5 using the BASEML program in the Phylogenetic Analysis by Maximum Likelihood (PAML) package [50]. The priors for the overall substitution rate were set at (a) G (1, 40.9) for the partial SSU dataset, (b) G (1, 30.9) for the SSU dataset, and (c) G (1, 34.3) for both of the LSU and SSU+LSU datasets. The prior for the rate-drift parameter was set at G (1, 2.32) for all the datasets. The independent-rates model [51] specified the prior rates among internal nodes. The parameters of the birth-death process for tree generation with species sampling [52] were fixed at λ = μ = 1 and ρ = 0. The root age and loose maximum bound for the root were set at 2.32 (23.2 Ma) and <3.72 (37.2 Ma) based on the FAD of *Neogloboquadrina continuosa* and *Paragloborotalia pseudokugleri*, which are the ancestral lineages of *P*. *obliquiloculata* + *N*. *dutertrei* and all three morphospecies studied including *G*. *inflata* [23] (Fig 3A). A hard and softbound version was used so that the probability of the true divergence time falls between the minimum and maximum bounds. The calibration nodes with minimum and maximum bounds assumed a heavy-tailed density based on a truncated Cauchy distribution of *p* = 0.1 and c = 1 as the default [50].

The MCMC approximation was obtained from a total of 1 × 10^4^ samples, which were taken every 20 cycles after a burn-in period of 5 × 10^4^ cycles. Two replicate MCMC runs with two different random seeds for each analysis were performed to confirm the convergence of the Markov chains to the stationary distribution. The MCMC samples from the two runs were combined after checking the distributions of the parameter values using the Tracer v 1.5 program [53]. The number of samples was large enough to reach the effective sample sizes (ESS > 200) for all parameters estimated in the present study.

## Results

### Single-gene and two-gene phylogenies

**Fig 4:**
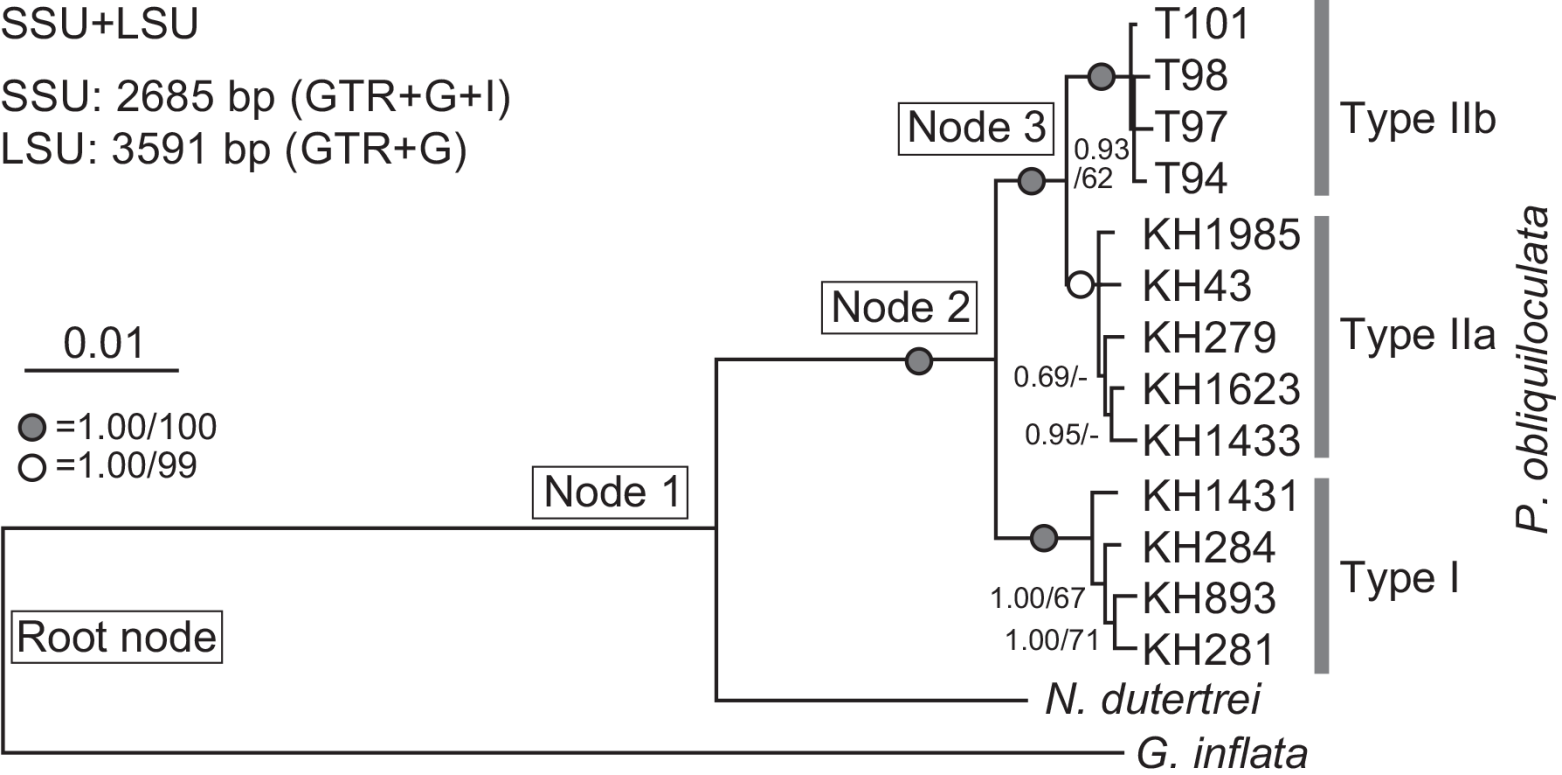
Bayesian phylogeny of the SSU + LSU rDNA sequences obtained from *P*. *obliquiloculata* and two outgroup species. Bayesian posterior probability (PP) and bootstrap values (BV) are shown at each node. The nodes used for divergence time estimations are named in enclosed boxes. Grey and white circles indicate high posterior PPs and BVs (PP/BV = 1.00/100 and PP/BP = 1.00/99, respectively).

Nearly complete SSU and LSU rDNA sequences were obtained from three morphospecies, *P*. *obliquiloculata*, *N*. *dutertrei*, and *G*. *inflata*. We confirmed that the LSU rDNA sequences in the present study branched from the published sequences of benthic foraminifers among the eukaryotes (data not shown). The phylogenies obtained from the two single-gene (SSU rDNA and LSU rDNA) datasets (S1 Fig) and the two-gene (SSU + LSU) dataset (Fig 4) showed the same topology, with three robust monophyletic clades associated with the three genetic types (Types I, IIa, and IIb) of *P*. *obliquiloculata*. The monophyletic clades based on the two-gene phylogeny showed the highest support levels (posterior probability of 1.00, bootstrap values of 99–100%).

### Estimation of divergence times

The estimated ages and CIs were configured for assessing the prior probability at each node. The estimated ages were 27.0–24.7, 11.0–8.9, 4.2–3.1, and 2.1–1.2 Ma at Root Node, Node 1, Node 2, and Node 3, respectively (Table 3, Fig 3B). The differences between these ages at Root Node and Node 1 were relatively larger (~2.0 Ma) than those at Nodes 2 and 3 (~1.0 Ma). In the same fossil calibration set, the ages at each node ranged within 0.7 Ma among the four gene-datasets, except for the age at Root Node in the set-4 (Table 3).

**Table 3:** List of divergence times estimated by using the four datasets: partial SSU, LSU, SSU, and SSU + LSU, with four different fossil calibration sets.

Node	Dataset		Set 1	Set 2	Set 3	Set 4	Diff.
	**Gene**						
**Root**	**partial SSU**	age	25.2	26.4	26.5	26.6	1.4
		CI (width)	37.4–11.3 (26.1)	37.6–12.7 (24.9)	37.4–13.4 (24.0)	36.9–18.4 (18.5)	(7.6)
	**LSU**	age	25.6	27.0	27.0	26.9	1.4
		CI (width)	37.6–12.1 (25.5)	37.7–13.5 (24.2)	37.3–14.0 (23.3)	36.8–18.4 (18.4)	(7.1)
	**SSU**	age	24.9	26.6	26.5	25.2	1.7
		CI (width)	37.4–12.5 (24.9)	37.6–14.3 (23.3)	37.3–14.4 (22.9)	36.0–18.2 (17.8)	(7.1)
	**SSU+LSU**	age	25.1	26.6	26.7	24.7	2.0
		CI (width)	37.3–13.8 (23.5)	37.5–15.6 (21.9)	37.3–15.9 (21.4)	34.6–18.3 (16.3)	(7.2)
	**Diff.**	age	0.7	0.6	0.5	2.2	
		width	(2.6)	(3.0)	(2.6)	(2.2)	
**1**	**partial SSU**	age	9.8	11.0	11.0	9.0	2.0
		CI (width)	18.8–5.6 (13.2)	20.1–6.7 (13.4)	20.1–6.7 (13.4)	10.9–6.8 (4.1)	(9.3)
	**LSU**	age	9.6	10.6	10.7	8.9	1.8
		CI (width)	18.0–5.6 (12.4)	18.9–6.7 (12.2)	18.8–6.7 (12.1)	10.9–6.8 (4.1)	(8.3)
	**SSU**	age	9.8	10.9	10.8	9.1	1.8
		CI (width)	16.9–5.6 (11.3)	18.1–6.7 (11.4)	17.9–6.7 (11.2)	10.9–6.9 (4.0)	(7.4)
	**SSU+LSU**	age	9.7	10.5	10.5	9.2	1.3
		CI (width)	15.8–5.6 (10.2)	16.1–6.7 (9.4)	16.3–6.7 (9.6)	10.9–6.9 (4.0)	(6.2)
	**Diff.**	age	0.2	0.5	0.5	0.3	
		width	(3.0)	(4.0)	(3.8)	(0.1)	
**2**	**partial SSU**	age	3.8	4.2	4.1	3.5	0.7
		CI (width)	9.0–1.1 (7.9)	10.0–1.3 (8.7)	9.6–1.3 (8.3)	7.0–1.3 (5.7)	(3.0)
	**LSU**	age	3.9	4.2	4.2	3.7	0.5
		CI (width)	8.3–1.6 (6.7)	8.7–1.9 (6.8)	8.7–1.9 (6.8)	6.3–1.9 (4.4)	(2.4)
	**SSU**	age	3.4	3.8	3.8	3.3	0.5
		CI (width)	6.6–1.5 (5.1)	7.3–1.8 (5.5)	7.3–1.8 (5.5)	5.4–1.9 (3.5)	(2.0)
	**SSU+LSU**	age	3.3	3.5	3.6	3.1	0.5
		CI (width)	5.7–1.7 (4.0)	5.8–2.0 (3.8)	5.9–2.0 (3.9)	4.6–2.1 (2.5)	(1.5)
	**Diff.**	age	0.6	0.7	0.6	0.6	
		width	(3.9)	(4.9)	(4.4)	(3.2)	
**3**	**partial SSU**	age	1.4	1.6	1.6	1.4	0.2
		CI (width)	4.1–0.2 (3.9)	4.5–0.2 (4.3)	4.5–0.2 (4.3)	3.5–0.1 (3.4)	(0.9)
	**LSU**	age	1.9	2.1	2.1	1.9	0.2
		CI (width)	4.6–0.7 (3.9)	4.9–0.8 (4.1)	4.9–0.8 (4.1)	3.6–0.8 (2.8)	(1.3)
	**SSU**	age	1.3	1.4	1.4	1.2	0.2
		CI (width)	2.7–0.4 (2.3)	3.1–0.5 (2.6)	3.0–0.5 (2.5)	2.4–0.5 (1.9)	(0.7)
	**SSU+LSU**	age	1.4	1.5	1.5	1.4	0.1
		CI (width)	2.6–0.7 (1.9)	2.7–0.8 (1.9)	2.7–0.7 (2.0)	2.2–0.8 (1.4)	(0.6)
	**Diff.**	age	0.6	0.7	0.7	0.7	
		width	(2.0)	(2.4)	(2.3)	(2.0)	

Age in Ma, CI: 95% credible interval in Ma, Diff.: Differences of the ages and widths among data and calibration sets.

**Fig 5:**
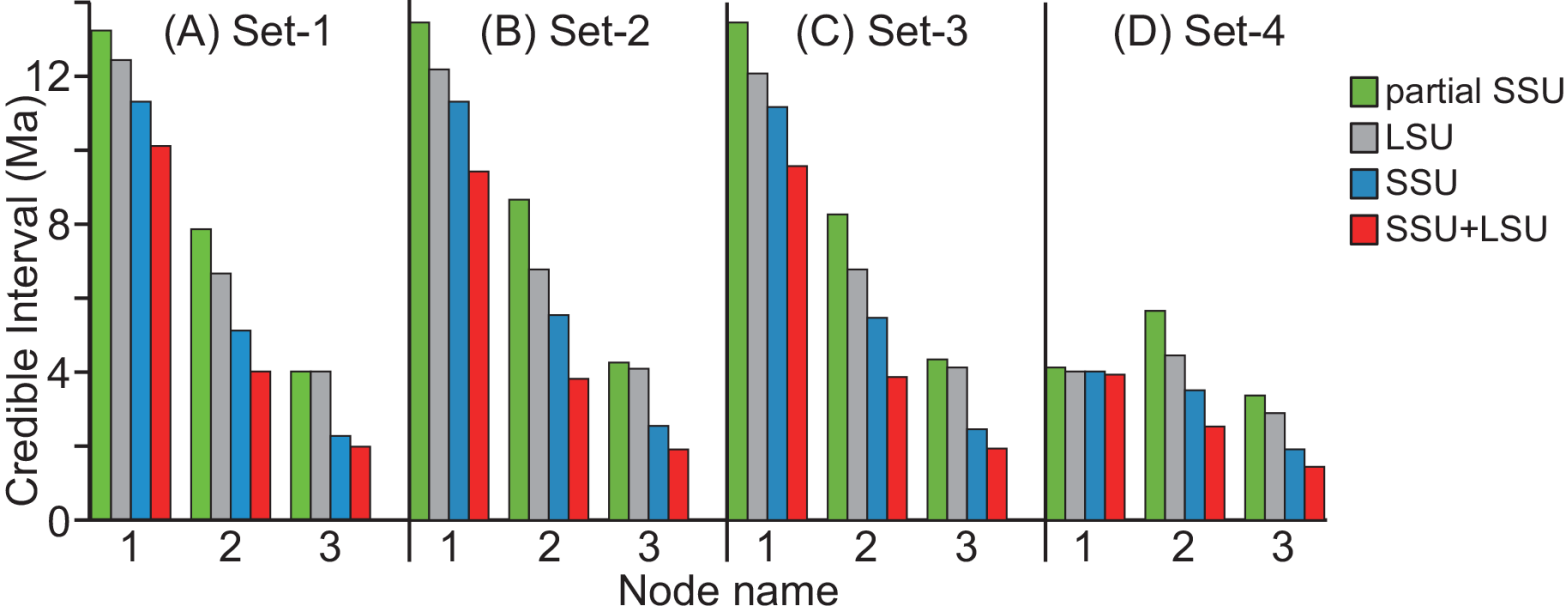
**The 95% credible intervals (CIs) at nodes 1, 2 and 3 obtained by divergence time estimations, based on the four gene datasets; partial SSU, LSU, SSU, and SSU+LSU, with four fossil calibration sets (A-D).** Node names correspond to Fig 3B.

In all estimations, the widths of CIs were found to be large in the partial SSU dataset, whereas they were found to be the smallest in the SSU+LSU dataset (Table 3, Fig 5). The CI widths tended to shorten gradually from the partial SSU, LSU, SSU to SSU+LSU datasets. Comparing the four fossil calibration sets, the largest differences (4.9 and 2.4 Ma) of the CI widths among genes at Nodes 2 and 3 were observed in set-2, whereas the smallest differences (3.2 and 2.0 Ma at Node 2 and 3) were found in set-4 (Table 3). The shortest widths of CIs at Nodes 2 and 3 were 2.5 and 1.4 Ma in the SSU+LSU dataset in the calibration set-4.

## Discussion

### Increasing phylogenetic resolution

All the phylogenies obtained in the present study clearly showed the presence of three genetically isolated species: the genetic types I, IIa, and IIb, in the morphospecies *P*. *obliquiloculata* (Fig 4, S1 Fig). In the previous study, the phylogeny based on the partial SSU rDNA sequences, corresponding with the helices 34–44 [36], showed that the Type IIb clade nested within a multidivergent clade of Type IIa [5]. In contrast, the phylogenies based on either the almost-complete SSU rDNA or LSU rDNA sequences showed the robust topology with distinct clades and appropriate phylogenetic relationships between the types IIb and IIa (S1 Fig). The combined analysis of the two genes increased the posterior probabilities and the bootstrap values (Fig 4). Therefore, we conclude that the increase in the number of nucleotide bases and genes is effective in elucidating the phylogenetic relationship between the species lineages.

The results of the divergence time estimations using four datasets showed that the width of CIs was shortened with the increase in nucleotide bases and genes (Fig 5, Table 3). In particular, the CI widths at Nodes 2 and 3 with the two-gene dataset were less than half of those with the partial SSU dataset, which has been used for the divergence time estimation in the previous studies [24,34,35]. The fossil calibrations also helped in achieving the precise estimation of the divergence times. This can be shown through the smallest width of CIs at Node 1 in the calibration set-4, in which both maximum and minimum time constraints restricted the prior probability distribution of Node 1 (Table 2, Fig 5). This strict calibration set affected the shortening of the CI widths at Nodes 2 and 3, as well (Fig 5). However, the CI widths have larger differences ranging from 3.2–4.9 and 2.0–2.4 Ma at Nodes 2 and 3 among the gene datasets than among the four calibration sets (1.5–3.0 and 0.6–1.3 Ma, respectively). This indicates that the precision of the divergence time estimation, in particular of the genetic types, is raised with increase in nucleotide bases and genes rather than by using the strict calibration set.

The estimated divergence ages of Nodes 2 and 3 were found to be slightly different either among the four calibration sets (0.1–0.7 Ma differences) or the four gene datasets (0.6–0.7 Ma differences) (Table 3). The estimated age varied slightly even if tight or relaxed calibrations were used. On the other hand, the ages estimated by the LSU rDNA dataset were relatively older than those estimated by the SSU rDNA dataset. These differences between the two single-gene datasets could be caused by the different evolutionary rates of genes [37]. However, all the estimated ages were within the CIs of SSU+LSU dataset in the most restricted calibration set-4 (Table 3). The use of at least two different genes in addition to appropriate fossil calibration is crucial to obtain precise estimates of divergence times of planktonic foraminiferal genetic types. According to the SSU+LSU dataset with the calibration set-4, the lineage *P*. *obliquiloculata* diverged into Types I and II around 3.1 Ma during the Pliocene, and the lineage Type II split into Types IIa and IIb around 1.4 Ma in the Pleistocene.

### Planktonic foraminiferal evolution and its relation to the ocean environmental changes

Recent phylogeographic studies have shown that the three genetic types (Types I, IIa, and IIb) of *P*. *obliquiloculata* are distributed from 24°13´S to 36°23´N of the world’s oceans [5,28] (Fig 1). The genetic type I is widely found from the tropical to the temperate areas of the Atlantic, Indian, and Pacific Oceans, whereas Type IIa is found only in the Western Pacific Warm Pool (WPWP), which is the largest and warmest area in the world’s oceans. The genetic type IIb is mainly found along the subtropical gyre margin of the Pacific Ocean, although one specimen was reported in the tropical Atlantic. Each of these genetic types is distributed in the mixed layer upper thermocline without any vertical separation [5]. All three types co-occur in the same water column in the WPWP. In the fossil record, the occurrence of the genus *Pulleniatina* has been found between 36.5°S and 41°N of the world’s oceans since ~7.0 Ma, as noted in the CHRONOS database (http://www.chronos.org). Since the appearance of *P*. *obliquiloculata*, its habitat has been restricted to the warm waters ranging from the tropical to temperate zones.

The estimation of divergence times from the molecular data suggest that the *P*. *obliquiloculata* lineage diverged twice, once at around 3.1 Ma during the late Pliocene and then at around 1.4 Ma during the middle Pleistocene (Fig 3B). The ocean environment was in the warm state from the Miocene to early Pliocene, in particular, the sea-surface temperature in the equatorial areas was higher than the present state [12,13,18,54–56]. In the Pacific, the WPWP was expanded in the latitudinal direction [54,55]. Moreover, high subsurface temperatures with a small east-to-west gradient occurred across the equator similar to the El-Niño event, although the depth of thermocline was entirely deeper than that of the modern El-Niño state [18]. The development of the warm ocean might have generated the wide and deep habitats for the warm marine species. Indeed, a high diversity of tropical coastal taxa (e.g. large benthic foraminifera, gastropods, fish, and corals) existed in the tropical oceans from the Miocene to Pliocene [10,57,58]. The warm state in the Pliocene possibly affected the expansion of habitat range for the pelagic species including *P*. *obliquiloculata*. However, the environmental conditions have shifted from the warm to cold states around ~4 Ma, owing to the tectonic changes with the closure of the Panama Seaway [14] and the growth of the Indonesian Archipelago [15], and the atmospheric-oceanic changes [17,18]. This cooling involved a change in the water column structure by shoaling and cooling of the equatorial thermocline [18,56]. These late Pliocene changes steepened the environmental gradient towards the margins of the warm water area, which was the habitat of the warm water species. The tropical organisms such as *P*. *obliquiloculata* that once extended their habitats toward the higher latitude areas during the warm period, were confronted with different environmental conditions in their marginal habitat from the tropics in the late Pliocene.

After the Pliocene, the Walker circulation promoted the growth of Northern Hemisphere ice sheets [15]. The development of Northern Hemisphere glaciation was associated with an increase in the latitudinal temperature gradient, which could be one of the driving forces for strengthening the subtropical gyres [16]. Subsequently, large glacial/interglacial oscillations have occurred around 1.7 Ma [16,17]. Along with the subtropical gyre, the western boundary currents, such as the Kuroshio Current, transfer the ocean heat from the equator to higher latitudinal zones. However, the impact of the subtropical gyre and its surface current system weakened during the glacial period, and then the environment of the higher latitudinal zones (subtropical to temperate areas) tended to cool [59]. Following this latitudinal shift of the warm water, the tropical pelagic plankton *P*. *obliquiloculata* might have been able to migrate in the latitudinal direction during the interglacial period, whereas it was exposed to the steep environmental gradient during the glacial periods. Consequently, both divergences around 3.1 and 1.4 Ma probably coincided with steepening of the environmental gradient from the tropical to temperate oceans but not with any geographic separation between the ocean basins and/or areas, where *P*. *obliquiloculata* is distributed. Environmental diversification in habitat might cause ecological differences that could trigger divergent selection and adaptive differentiation in individuals leading to reproductive isolation [8,60]. The *P*. *obliquiloculata* population across the steep environmental gradient might have been ecologically differentiated between the core (tropical) and marginal (temperate) area. Such ecological changes may have forced the organisms to adapt to new environments if they were to survive. Although future studies are needed to investigate the ecological differences among the three genetic types of *P*. *obliquiloculata*, they have diverged when adaptive differentiation caused reproductive isolation between the core and marginal habitats.

The geographic distributions and divergence ages of *P*. *obliquiloculata* genetic types help in hypothesizing their evolutionary history, though it is impossible at the present moment to assign which oceans were their origins. The genetic type IIb is found in the tropical Atlantic and the Pacific, despite its divergence after the complete closure of the Panama Seaway between two oceans (Figs 1 and 3B). One possibility is that Type IIb could have migrated across the Pacific-Indian-Atlantic ocean basins through the global ocean circulation. However, no specimens of Type IIb were found in the Indian Ocean despite the thorough surveys [5,28] (Fig 1), which indicates a low possibility for inter-basin transfer through global ocean circulation. Another plausible possibility could be that Type IIb is the descendant of the Type II lineage, which is globally distributed in the Pacific and Atlantic Oceans. The Panama Seaway was still open around 3.1 Ma, when the lineages of Types I and II diverged (Fig 3B). The eastward-flowing and westward-flowing currents facilitated the dispersal of marine taxa between the Atlantic and East Pacific Oceans until ~2.6 Ma [57,61,62]. The populations of Type II might have remained in both oceans as the Type IIb lineage. Following this scenario, Type IIa diverged from the Type IIb lineage around 1.4 Ma. This young lineage have had narrow habitat range with temperature ranging between 25.4 and 29.2°C, compared with Type IIb having a wider temperature range between 13.9 and 29.2°C [5]. Taking into account the later divergence of Type IIa and its biogeography, Type IIa was considered as the residual population in the stable and warm areas of the tropical ocean. On the other hand, Type IIb could be the remaining survivor owing to the ecological differences generated among Type II populations along the sharp gradient in the warm waters.

The present study demonstrates that increasing the number of nucleotide bases of phylogenetically useful genes enables a precise estimation of the divergence times of planktonic foraminiferal genetic types. The genetic divergence of *P*. *obliquiloculata* has not caused by geographic (physical) separation but rather seems to be associated with ocean environmental changes in the warm water areas from the Pliocene to Pleistocene. An increase in precision of the divergence time estimation thus not only resolves the relationship between the evolutionary history and paleoenvironmental changes, enhancing our understanding of how the evolution of organisms is associated with environments.

## Supporting Information

S1 Fig Bayesian phylogenies of the SSU rDNA (A) and LSU rDNA (B) sequences obtained for *P*. *obliquiloculata* and two outgroup species. Numbers and circles on the nodes are in the same manner with Fig 4.
(EPS)

## References

[1] Caron DA, Worden AZ, Countway PD, Demir E, Heidelberg KB. Protists are microbes too: a perspective. ISME J. 2009; 3:4–12. 10.1038/ismej.2008.101 19005497

[2] Barton AD, Dutkiewicz S, Flierl G, Bragg J, Follows MJ. Pattern of diversity in marine phytoplankton. Science 2011; 327: 1509–1511. 10.1126/science.1184961 20185684

[3] Aurahs R, Grimm GW, Hemleben V, Hemleben C, Kucera M. Geographic distribution of cryptic genetic types in the planktonic foraminifer *Globigerinoides ruber*. Molecular Ecology 2009; 18: 1692–1706. 10.1111/j.1365-294X.2009.04136.x 19302352

[4] Casteleyn G, Leliaert F, Backeljau T, Debeer AE, Kotaki Y, Rhodes L, et al Limits to gene flow in a cosmopolitan marine planktonic diatom. Proc Natl Acad Sci USA 2011; 107: 12952–12957. 10.1073/pnas.1001380107 PMC2919969 20615950

[5] Ujiié Y, Asami T, de Garidel-Thoron T, Liu H, Ishitani Y, de Vargas C. Longitudinal differentiation among pelagic populations in a planktic foraminifer. Ecology and Evolution 2012; 2: 1725–1737. 10.1002/ece3.286 22957176 PMC3434911

[6] Ishitani Y, Ujiié Y, Takishita K. Uncovering sibling species in Radiolaria: Evidence for ecological partitioning in a marine planktonic protest. Molecular Phylogenetics and Evolution 2014; 78: 215–222. 10.1016/j.ympev.2014.05.021 24862224

[7] Peijnenburg KTCA, Goetze E. High evolutionary potential of marine zooplankton. Ecology and Evolution 2013; 3: 2765–2781. 10.1002/ece3.644 24567838 PMC3930040

[8] Nosil P. Ecological Speciation. Oxford, UK: Oxford University Press; 2012.

[9] Rutherford S, D’Hondt S, Prell W. Environmental controls on the geographic distribution of zooplankton diversity. Nature 1999; 400: 749–753.

[10] Renema W, Bellwood DR, Braga JC, Bromfield K, Hall R, Johnson KG, et al Hopping Hotspots: Global Shifts in Marine Biodiversity. Science 2008; 321: 654–657. 10.1126/science.1155674 18669854

[11] Tittensor DP, Mora C, Jetz W, Lotze HK, Ricard D, Berghe EV, et al Global patterns and predictors of marine biodiversity across taxa. Nature 2010; 466: 1098–1101. 10.1038/nature09329 20668450

[12] Fedorov AV, Brierley CM, Lawrence KT, Liu Z, Dekens PS, Ravelo AC. Patterns and mechanisms of early Pliocene warmth. Nature 2013; 496: 43–49. 10.1038/nature12003 23552943

[13] O’Brien CL, Foster GL, Martínez-Botí MA, Abell R, Rae JWB, Pancost RD. High sea surface temperatures in tropical warm pools during the Pliocene. Nature Geoscience 2014; 7: 606–611.

[14] Lunt DJ, Foster GL, Haywood AM, Stone EJ. Late Pliocene Greenland glaciation controlled by a decline in atmospheric CO2 levels. Nature 2008; 454: 1102–1105. 10.1038/nature07223 18756254

[15] Molnar P, Cronin TW. Growth of the Maritime Continent and its possible contribution to recurring ice ages. Paleoceanography 2015; 30: 196–225.

[16] Ravelo AC, Andreasen DH, Lyle M, Lyle AO, Wara MW. Regional climate shifts caused by gradual global cooling in the Pliocene epoch. Nature 2004; 429: 263–267. 15152244 10.1038/nature02567

[17] Wara MW, Ravelo AC, Delaney ML. Permanent El Niño–Like Conditions During the Pliocene Warm Period. Science 2005; 309: 758–761. 15976271 10.1126/science.1112596

[18] Ford HL, Ravelo AC, Dekens PS, LaRiviere JP, Wara MW. The evolution of the equatorial thermocline and the early Pliocene El Padre mean state. Geophys Res Lett. 2015; 42: 4878–4887.

[19] Leigh EG, O’Dea A, Vermeij GJ. Historical biogeography of the Isthmus of Panama. Biological Reviews 2015; 89: 148–172. 10.1111/brv.12048 23869709

[20] Tornabene L, Valdez S, Erdmann M, Pezold F. Support for a ‘Center of Origin’ in the Coral Triangle: Cryptic diversity, recent speciation, and local endemism in a diverse lineage of reef fishes (Gobiidae: Eviota). Molecular Phylogenetics and Evolution 2015; 82: 200–210. 10.1016/j.ympev.2014.09.012 25300452

[21] Morard R, Darling KF, Mahé F, Audic S, Ujiié Y, Weiner AKM, et al PFR^2^: a curated database of planktonic foraminifera 18S ribosomal DNA as a resource for studies of plankton ecology, biogeography and evolution. Molecular Ecology Resources 2015; 15: 1472–1485. 10.1111/1755-0998.12410 25828689

[22] Berggren WA, Hilgen FJ, Langereis CG, Kent DV, Obradovich JD, Raffi I, et al Late Neogene chronology: new perspectives in highresolution stratigraphy. Geol Soc Am Bull. 1995; 107:1272–1287.

[23] Aze T, Ezard THG, Purvis A, Coxall HK, Stewart DRM, Wade BS, et al A phylogeny of Cenozoic macroperforate planktonic foraminifera from fossil data: Biological Reviews 2011; 86: 900–927. 10.1111/j.1469-185X.2011.00178.x 21492379

[24] Darling KF, Kucera M, Pudsey CJ, Wade CM. Molecular evidence links cryptic diversification in polar planktonic protists to Quaternary climate dynamics. Proc Natl Acad Sci USA 2004; 101: 7657–7662. 15136732 10.1073/pnas.0402401101 PMC419662

[25] Ujiié Y, Lipps JH. Cryptic diversity in planktonic foraminifera in the northwest Pacific Ocean. Journal of Foraminiferal Research 2009; 39: 145–154.

[26] Bé AWH. An ecological, zoogeographical and taxonomic review of Recent planktonic foraminifera In: Ramsay ATS, editor. Oceanic Micropaleontology. United States: Academic Press; 1977 p. 1–100.

[27] Kennett JP, Srinivasan S. Neogene planktonic foraminifera United Kingdom: Hutchinson Ross Pub. Co; 1983.

[28] Seears H, Darling K, Wade C. Ecological partitioning and diversity in tropical planktonic foraminifera. BioMedical Center Evolutionary Biology 2012; 12:54. 10.1186/1471-2148-12-54 PMC3361484 22507289

[29] Locarnini RA, Mishonov AV, Antonov JI, Boyer TP, Garcia HE. World Ocean Atlas 2005, vol. 1, Temperature, NOAA Atlas NESDIS, vol. 61, edited by Levitus S.. Md: NOAA, Silver Spring; pp.182.

[30] Schlitzer R. Ocean Data View; 2015. Available: http://odv.awi.de. Accepted on October, 2015.

[31] de Vargas C, Norris R, Zaninetti L, Gibb SW, Pawlowski J. Molecular evidence of cryptic speciation in planktonic foraminifers and their relation to oceanic provinces: Proc Natl Acad Sci USA 1992; 96: 2864–2868. 10.1073/pnas.96.6.2864 PMC15860 10077602

[32] de Vargas C, Pawlowski J. Molecular versus taxonomic rates of evolution in planktonic foraminifera: Molecular Phylogenetics and Evolution 1998; 9: 463–469. 9667994 10.1006/mpev.1998.0491

[33] Douzery EJP, Snell EA, Bapteste E, Delsuc F, Philippe H. The timing of eukaryotic evolution: does a relaxed molecular clock reconcile proteins and fossils? Proc Natl Acad Sci USA 2004; 101: 15386–15391. 15494441 10.1073/pnas.0403984101 PMC524432

[34] Aurahs R, Treis Y, Darling K, Kucera M. A revised taxonomic and phylogenetic concept for the planktonic foraminifer species *Globigerinoides ruber* based on molecular and morphometric evidence. Marine Micropaleontology 2011; 79: 1–14.

[35] Weiner AKM, Weinkauf MFG, Kurasawa A, Darling KF, Kucera M, Grimm GW. Phylogeography of the Tropical Planktonic Foraminifera Lineage *Globigerinella* Reveals Isolation Inconsistent with Passive Dispersal by Ocean Currents. PLoS ONE 2014; 9(3): e92148 10.1371/journal.pone.0092148 24663038 PMC3963880

[36] Pawlowski J, Lecroq B. Short rDNA barcodes for species identification in foraminifera. Journal of Eukaryotic Microbiology 2010; 57: 197–205. 10.1111/j.1550-7408.2009.00468.x 20113377

[37] Kumar S, Subramanian S. Mutation rates in mammalian genomes. Proc Natl Acad Sci USA 2002; 99: 803–808. 11792858 10.1073/pnas.022629899 PMC117386

[38] Flakowski J, Bolivar I, Fahrni J, Pawlowski J. Tempo and mode of spliceosomal intron evolution in actin of foraminifera. Journal of Molecular Evolution 2006; 63(1): 30–41. 16755352 10.1007/s00239-005-0061-z

[39] Ishitani Y, Ishikawa SA, Inagaki Y, Tsuchiya M, Takahashi K, Takishita K. Multigene phylogenetic analyses including diverse radiolarian species support the ‘‘Retaria” hypothesis–the sister relationship of Radiolaria and Foraminifera. Marine Micropaleontology 2011; 81: 32–42.

[40] Groussin M, Pawlowski J, Ziheng Y. Bayesian relaxed clock estimation of divergence times in foraminifera. Molecular Phylogenetics and Evolution 2011; 61:157–166. 10.1016/j.ympev.2011.06.008 21723398

[41] Pawlowski J, Bolivar I, Guiard-Maffia J, Gouy M. Phylogenetic position of foraminifera inferred from LSU rRNA gene sequences. Molecular Biology and Evolution 1994; 11: 929–938. 7815931 10.1093/oxfordjournals.molbev.a040174

[42] Gouy M, Guindon S, Gascuel O. SeaView version 4: A multiplatform graphical user interface for sequence alignment and phylogenetic tree building: Molecular Biology and Evolution 2010; 27: 221–224. 10.1093/molbev/msp259 19854763

[43] Schweizer M, Pawlowski J, Kouwenhoven TJ, Guiard J, van der Zwaan B. Molecular phylogeny of Rotaliida (Foraminifera) based on complete small subunit rDNA sequences. Marine Micropaleontology 2008; 66: 233–246.

[44] Nylander JAA.MrModeltest v2; 2004. Available: http://www.abc.se/~nylander/. Accessed on June, 2015.

[45] Jobb G. TREEFINDER version of October 2008; 2008. Available: http://www.treefinder.de Accessed on October, 2008.

[46] Yang Z. Maximum likelihood phylogenetic estimation from DNA sequences with variable rates over sites: approximate methods. Journal of Molecular Evolution1994a; 39: 306–314. 7932792 10.1007/BF00160154

[47] Yang Z. Estimating the pattern of nucleotide substitution. Journal of Molecular Evolution 1994; 39: 105–111. 8064867 10.1007/BF00178256

[48] Gu X, Fu Y-X, Li W-H. Maximum likelihood estimation of the heterogeneity of substitution rate among nucleotide sites. Molecular Biology and Evolution 1995; 12: 546–557. 7659011 10.1093/oxfordjournals.molbev.a040235

[49] Ronquist F, Huelsenbeck JP. MRBAYES 3: Bayesian phylogenetic inference under mixed models: Bioinformatics 2003; 19:1572–1574. 12912839 10.1093/bioinformatics/btg180

[50] Yang Z. PAML4: phylogenetic analysis by maximum likelihood: Molecular Biology and Evolution 2007; 24: 1586–1591. 17483113 10.1093/molbev/msm088

[51] Rannala B, Yang Z. Inferring speciation times under an episodic molecular clock. Systematic Biology 2007; 56: 453–466. 17558967 10.1080/10635150701420643

[52] Yang Z, Rannala B. Bayesian Phylogenetic Inference Using DNA Sequences: A Markov Chain Monte Carlo Method. Molecular Biology and Evolution 1997; 14: 717–724. 9214744 10.1093/oxfordjournals.molbev.a025811

[53] Drummond A, Rambaut A. Tracer; 2008. Available: http://tree.bio.ed.ac.uk/software/tracer. Accessed on December, 2013.

[54] Brierley CM, Fedorov AV, Liu Z, Herbert TD, Lawrence KT, Lariviere JP. Greatly expanded tropical warm pool and weakened Hadley circulation in the early Pliocene. Science 2009; 323(5922): 1714–1718. 10.1126/science.1167625 19251592

[55] LaRiviere JP, Ravelo AC, Crimmins A, Dekens PS, Ford HL, Lyle M, et al Late Miocene decoupling of oceanic warmth and atmospheric carbon dioxide forcing. Nature 2012; 486(7401): 97–100. 10.1038/nature11200 22678287

[56] Zhang YG, Pagani M, Liu Z. A 12-million-year temperature history of the tropical Pacific Ocean. Science 2014; 344: 84–87. 10.1126/science.1246172 24700856

[57] Duda TF Jr., Kohn AJ. Species-level phylogeography and evolutionary history of the hyperdiverse marine gastropod genus *Conus*. Molecular Phylogenetics and Evolution 2005; 34: 257–272. 15619440 10.1016/j.ympev.2004.09.012

[58] Postaire B, Bruggemann JH, Magalon H, Faure B. Evolutionary dynamics in the southwest Indian Ocean marine biodiversity hotspot: A perspective from the rocky shore gastropod genus *Nerita*. PLoS ONE 2014; 9(4): e95040 10.1371/journal.pone.0095040 24736639 PMC3988148

[59] Ujiié Y, Ujiié H, Taira A, Nakamura T, Oguri K. Spatial and temporal variability of surface water in the Kuroshio source region, Pacific Ocean, over the past 21,000 years: evidence from planktonic foraminifera. Marine Micropaleontology 2003; 49: 335–364.

[60] Funk WC, Murphy MA, Hoke KL, Muths E, Amburgey SM, Lemmon EM, et al Elevational speciation in action? Restricted gene flow associated with adaptive divergence across an altitudinal gradient. Journal of Evolutionary Biology 2015; 10.1111/jeb.12760 26363130

[61] Meyer CP. Molecular systematics of cowries (Gastropoda: Cypraeidae) and diversification patterns in the tropics. Biological Journal of the Linnean Society 2003; 79: 401–459.

[62] Herbert G. Systematic revision of the genus Eupleura H. and A. Adams, 1853 (Gastropoda: Muricidae) in the Neogene to recent of tropical America. Veliger 2005; 47: 294–331.

